# Identification of single major QTL and candidate gene(s) governing hull-less seed trait in pumpkin

**DOI:** 10.3389/fpls.2022.948106

**Published:** 2022-08-11

**Authors:** Barinder Kaur, Karmvir Singh Garcha, Dharminder Bhatia, Jiffinvir Singh Khosa, Madhu Sharma, Amandeep Mittal, Neha Verma, Ajmer Singh Dhatt

**Affiliations:** ^1^Department of Vegetable Science, Punjab Agricultural University, Ludhiana, Punjab, India; ^2^Department of Plant Breeding and Genetics, Punjab Agricultural University, Ludhiana, Punjab, India; ^3^School of Agricultural Biotechnology, Punjab Agricultural University, Ludhiana, Punjab, India; ^4^Directorate of Research, Punjab Agricultural University, Ludhiana, Punjab, India

**Keywords:** hull-less seed, *Cucurbita pepo*, BSA, QTL-seq, MAS

## Abstract

The hull-less pumpkin (*Cucurbita pepo*) seed does not require de-hulling before use for human consumption, as a result highly preferred by the oil, nut, and baking industries. In hull-less seeds, a single recessive gene is responsible for the absence of outer thick seed coat layers; however, the genomic region and gene(s) controlling the trait are unclear to date. In this study, four crosses attempted to derive F_2_ and backcross populations confirmed the single recessive gene inheritance of hull-less seed trait in populations adapted to the sub-tropical climate. The candidate genomic region for hull-less seed trait was identified through the BSA-QTLseq approach using bulks of F_2:3_ progenies from a cross of HP111 (hulled) and HLP36 (hull-less). A novel genomic region on chromosome 12 ranging from 1.80 to 3.86 Mb was associated with the hull-less seed trait. The re-sequencing data identified a total of 396 SNPs within this region and eight were successfully converted into polymorphic KASP assays. The genotyping of segregating F_2_ (*n* = 160) with polymorphic KASP assays resulted in a 40.3 cM partial linkage map and identified Cp_3430407 (10 cM) and Cp_3498687 (16.1 cM) as flanking markers for hull-less locus (*Cphl-1*). These flanking markers correspond to the 68.28 kb region in the reference genome, and the marker, Cp_3430407 successfully predicted the genotype in 93.33% of the *C. pepo* hull-less germplasm lines, thus can be used for marker-assisted selection in parents polymorphic for the hull-less seed trait. The *Cphl-1*-linked genomic region (2.06 Mb) encompasses a total of 182 genes, including secondary cell wall and lignin biosynthesis-related transcriptional factors *viz*., “NAC” (*Cp4.1LG12g04350*) and “MYB” (*Cp4.1LG12g03120*). These genes were differentially expressed in the seeds of hulled and hull-less genotypes, and therefore could be the potential candidate genes governing the hull-less seed trait in pumpkin.

## Introduction

Pumpkin (*Cucurbita pepo* L.) is an important vegetable crop that belongs to the Cucurbitaceae family, which is grown worldwide for its flesh and seeds. Pumpkin seeds are extensively used in the bakery and oil industry due to their high nutritional value (Jafari et al., 2012; De Lamo and Gómez, 2018). Proteins, unsaturated fatty acids (linoleic, oleic, and palmitic acids), minerals (potassium, phosphorous, and zinc), and vitamins (Vitamin E) are the key nutritionally important constituents found in the pumpkin seeds (Dotto and Chacha, 2020). The consumption of pumpkin seeds has positive effects on human health due to their anti-cancer, anti-diabetic, and anti-inflammatory properties (Fruhwirth and Hermetter, 2007; Stevenson et al., 2007; Yoshinari et al., 2009). However, the presence of a thick and leathery seed coat (hull) due to the strong lignification of testa layers requires de-hulling prior to use for human consumption, which proves to be one of the main hindrances in the wide acceptability of the pumpkin seeds (Lelley et al., 2009). A spontaneous mutant called Styrian (hull-less) (*C. pepo* subsp. *pepo* var. *styriaca*) was identified in Austria that lacks the hard seed coat, and as a result, does not require the expensive decortication process (Lelley et al., 2009). Histological characterization of hull-less pumpkin seeds reveals that seed coat development is similar in both hulled and hull-less seeds till 10 days post-anthesis, but after that extent of lignification decreases in hull-less seeds (Stuart and Loy, 1983; Bezold et al., 2005).

Over the years, several studies investigated the genetic inheritance of hull-less seed coat in pumpkins and concluded that a single recessive gene in the homozygous condition (*hh*) is responsible for the lack of a hard seed coat; however, reports of some modifiers having a minor influence on testa development are also available (Zraidi et al., 2003; Lelley et al., 2009). The availability of hull-less seed trait allowed the breeders to develop pumpkin cultivars for edible seed purposes, especially in Europe and North America (Winkler, 2000). A hull-less seeded variety “Lady Godiva” (EC 664187) has been introduced by Punjab Agricultural University (PAU), Ludhiana, India, and the trait has been transferred into adapted germplasm to release the first Indian hull-less seeded variety “*PAU Magaz Kadoo*-1” through conventional breeding approaches (Dhatt et al., 2020). However, the conventional breeding methods are time-consuming and labor intensive, which demands dexterous efforts for the identification of hull-less trait from segregating generations and transferring them into different genetic backgrounds. Marker-assisted selection (MAS) can overcome these hurdles but requires the development of tightly linked molecular markers with hull-less seed trait (Gong et al., 2008).

The decrease in the cost of whole-genome sequencing and technological advances in genomics led to the development of a large number of single nucleotide polymorphisms (SNPs) that allow researchers to identify genomic regions governing agronomically important traits more efficiently in comparison to traditional mapping approaches (Varshney et al., 2021). In different crops, it has been demonstrated that QTL-Seq analysis is a fast and efficient approach to identify candidate genomic regions and develop tightly linked SNPs to utilize in marker-assisted selection for traits of economic interest (Takagi et al., 2013; Lu et al., 2014; Illa-Berenguer et al., 2015). QTL-Seq is based on the combination of bulk segregant analysis (BSA) and next-generation sequencing (NGS), in which two extreme phenotypes with respect to the target trait are selected from a segregating population and bulked separately. These bulks are sequenced along with their parents and further subjected to downstream bioinformatic analysis to identify candidate genomic regions (Takagi et al., 2013).

In the present investigation, we studied the genetic inheritance of hull-less seed trait in pumpkin populations adapted to the sub-tropical climate and used QTL-Seq to identify the candidate genomic region responsible for hull-less seed. The genes present in the genomic region were analyzed, and the expression pattern of secondary cell wall/ lignin biosynthesis-related genes was studied in the seeds of hulled and hull-less genotypes at different time intervals (Wyatt et al., 2015). Furthermore, we developed the Kompetitive allele-specific PCR (KASP) markers and demonstrated their potential to follow marker-assisted selection for hull-less seed trait to expedite edible seeded pumpkin breeding programmes.

## Materials and methods

### Genetic inheritance pattern of hull-less seed trait

Three hull-less genotypes *viz*., HLP36, HLP53, and HLP8 and two hulled genotypes *viz*., HP111 and HP112 of *C. pepo* adapted to the sub-tropical climate of India were used. Crosses were attempted between HP111 × HLP36, HP111 × HLP53, HP112 × HLP8, and HLP8 × HP112 to develop F_1_, F_2_, BC_1_P_1_, and BC_1_P_2_ generations. Seeds of all generations of the above-mentioned cross combinations were sown during subsequent seasons and phenotyped for hulled or hull-less seeds, according to Zraidi et al. (2003). A Chi-square test was used to evaluate the goodness of fit with different testing ratios to determine the genetic control of the hull-less seed trait.

### Construction of extreme bulks for hull-less seed trait locus identification

**Figure 1:**
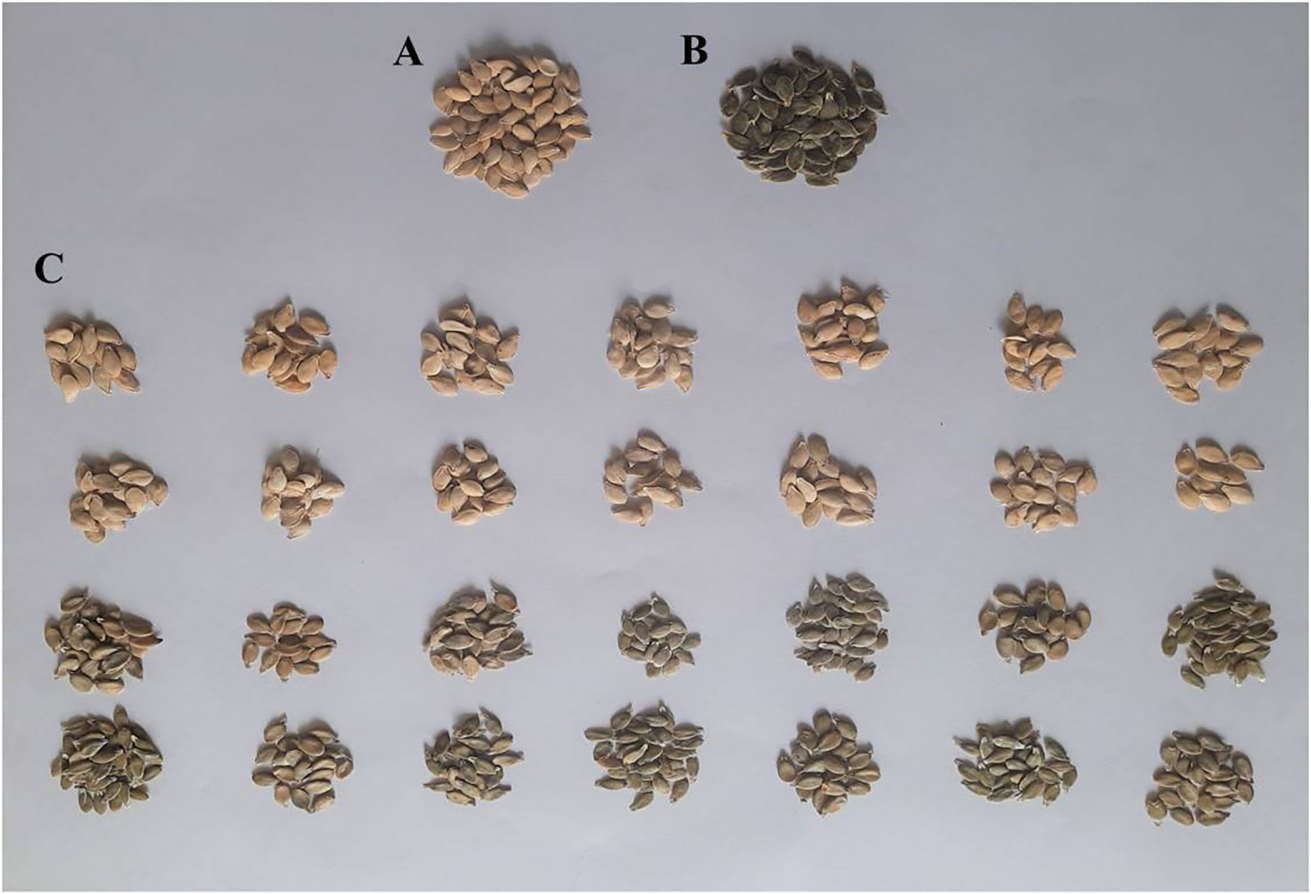
Seeds of *C. pepo* parents and bulks used in QTL-seq analysis. **(A)** Hulled Seeds of HP111; **(B)** Hull-less seeds of HLP36; **(C)** F_2:3_ hulled (1st and 2nd row) and hull-less bulks (3rd and 4th row) of HP111 × HLP36.

Total genomic DNA was extracted from young leaves of two parental lines *viz*., HP111 (hulled) and HLP36 (hull-less), and their F_2:3_ population using the CTAB method (Doyle and Doyle, 1987). For Bulked Segregant Analysis, hulled and hull-less seeded F_2:3_ progenies developed from HP111 × HLP36 along with their parental lines were used (Figure 1). Two DNA bulks were separately constructed by mixing an equal amount of DNA extracted from 14 extremely hulled (B_1_) and 14 hull-less seeded (B_2_) F_2:3_ progenies. Similarly, for parents, DNA from five plants was extracted and bulked. DNA quality was evaluated by running the samples on 0.8% agarose gel and was quantified using NanoDrop 8000 (Thermo Scientific Inc., USA), and the final DNA concentration was adjusted to 100 ng/μl. Subsequently, the genomic DNA of all the four samples (P_1_, P_2_, B_1_, and B_2_) were outsourced to the NGB Diagnostics Pvt Ltd., Noida, India, for whole genome re-sequencing using the Illumina HiSeq platform.

### QTL-seq to identify the genomic region governing hull-less seed trait

QTL-seq pipeline (QTL-seq_framework1.4.4) was used for the mapping of QTL(s) for hull-less seed trait (Takagi et al., 2013). The raw paired-end sequencing reads were filtered with Phred quality scores (*q*) < 30 and quality threshold value (*p*) < 90. Then, the clean reads of both the parents were aligned to the publically available reference genome of *C. pepo* cv. Zucchini (Montero-Pau et al., 2017) using BWA (Burrows-Wheeler Alignment Tool) version 0.5.9 (Li and Durbin, 2009). Alignment files were converted to SAM/BAM files, and SNPs were identified with Samtools version 0.1.8 (Li et al., 2009) and refined with the Coval software (Kosugi et al., 2013). Finally, the reference-guided parent assemblies were developed for hulled and hull-less parents by substituting the reference bases with alternative bases at the positions of confidence SNPs in the genomic sequences of *C. pepo*. To identify the genomic regions controlling hull-less seed trait, short reads of both the bulks were first aligned to reference guided parent assemblies, and then SNPs were called. The SNP calling filter, Coval v1.4.1 (cov), was set to a mutation index of 2, 3, and 4 with a reading depth threshold (co) of 5 and 7 to improve the accuracy. SNP-index was computed for both the bulks at each SNP position as a proportion of reads harboring the SNP that are dissimilar from the reference sequence. If all reads matched the reference, the SNP index was 0, and 1 if all reads were different than the reference allele.

Afterwards, the ΔSNP index was calculated for each position as the SNP index of one bulk was subtracted from the SNP index of the other. Sliding window analysis was conducted with 2 and 4 Mb intervals and 50 kb increment, and ΔSNP-index plots were constructed w.r.t confidence intervals (95% and 99%) under the null hypothesis of no QTL. Plots are represented using 2 Mb window size, 50 kb increment with cov 2 and co 7. Only SNPs with ΔSNP index significantly higher than 0.5 or lower than −0.5 at the 95 and/or 99% confidence level were considered as the effective SNPs for hull-less seed trait. Furthermore, the QTLseqr pipeline was also used to calculate *p*-values at an FDR (*q*) of 0.001 to identify potential QTLs associated with the hull-less seed trait (Mansfeld and Grumet, 2018). In this approach, variants were called using GATK (Genome Analysis Toolkit) version 4.1.4.1 'HaplotypeCaller' (Van der Auwera et al., 2013) and then filtered according to read depth (>5) and genotype quality (>30) with vcftools version 0.1.13. Further filtering of SNPs was performed using *filterSNPs()* function based on reference allele frequency = 0.10, minTotalDepth ≥ 10, maxTotalDepth ≤ 400, minSampleDepth ≥14 and genotype quality score (GQ ≥ 30).

### Validation of candidate region controlling hull-less seed trait using kompetitive allele-specific PCR assays

Polymorphic SNPs were identified from the candidate genomic region for hull-less seed trait on the basis of high delta SNP index and parental polymorphism. Each SNP site was parsed to retrieve the up- and downstream sequences of 100 bp in the target region using samtools (Semagn et al., 2014) with a criterion of no other SNP and InDels in visualization *via* IGV software **(**https://software.broadinstitute.org/software/igv/). For each selected SNP, two allele-specific forward primers and one common reverse primer were designed using the Primer3 software with selection on the basis of relatively unique primer sequences in the *C. pepo* genome. Target SNPs were used to develop 21 KASP assays (Supplementary Table 1) to validate the candidate genomic region governing the hull-less seed trait.

PCR amplifications were performed using a Veriti 384-Well Thermal Cycler (Applied Biosystems) in a 4 μL reaction volume, comprised of 2 μL of 100 ng/μL genomic DNA, 1.946 μL 2 × low rox KASP™ master mix (LGC Genomics LLC) and 0.056 μL of primer mix. The PCR conditions were set at 95°C for 15 min, followed by touchdown PCR of 10 cycles at 95°C for 20 s, 66°C (reduced by 0.6 °C per cycle) for 25 s, and 72°C for 15 s; subsequently, 35 cycles of 10 s at 95°C, 1 min at 60°C, 15 s at 72°C, and a final extension at 72°C for 5 min. Fluorescent endpoint readings were recorded using an Infinite M200Pro plate reader (Tecan, Group Ltd.), and genotyping calls were made using KlusterCaller™ (LGC Genomics LLC). The fluorescent readings for genotyping a few individuals were ambiguous and considered as missing data that was not included in linkage map construction and marker accuracy prediction.

### Linkage analysis, candidate gene identification, and functional annotation

All 21 KASP assays were used to genotype both hulled and hull-less parents along with F_1_ hybrid plants. The parental polymorphic KASP assays were used to genotype F2:3 progenies of hulled and hull-less seeded used in bulk construction and 160 F_2_ individuals derived from the HP111 × HLP36 cross. A partial genetic linkage was developed with Join Map 4.1 using the Kosambi mapping function and an LOD score of 5 (Van Ooijen, 2006). The final genetic map was drawn in the MapChart software (Voorrips, 2002).

The linked KASP assays were also tested on the diversity panel using 45 diverse hulled and hull-less genotypes of *C. pepo*. For the identification of candidate genes, the protein sequences of all the genes present in the QTL-seq identified region were evaluated for their homology with *Arabidopsis thaliana* (TAIR10) genes involved in the cell wall, cellulose, and lignin biosynthesis.

### Expression analysis of genes present in the candidate genomic region and time course differential RNA seq expression between hulled and hull-less seeded genotypes

The expression of genes present in the candidate genomic region was estimated using a publically available RNA-seq dataset [Cucurbit Genomics Database (http://cucurbitgenomics.org/organism/14)] over different developmental stages of hulled and hull-less seeds [at 5, 10, 15, 20, and 40 days after pollination (DAP)] (Wyatt et al., 2015). The time course differential RNA seq expression was used for the differential expression between seeds of hulled (Sweet Reeba) and hull-less (Lady Godiva) genotypes at 15 and 20 DAP, and a total number of differentially expressed genes was calculated using inbuilt edgeR based algorithms with adjusted *p*-value cutoff at 0.05 and fold change cutoff at 4. Furthermore, gene ontology analysis (Biological processes) with a *p*-value as good or better than 0.05 was used to calculate gene ontology terms related to cell wall biosynthesis or lignin biosynthesis.

## Results

### Genetic inheritance pattern of hull-less seed trait in segregating populations

In our breeding programme, hull-less seed trait from the variety Lady Godiva has been transferred into local *C. pepo* lines adapted to the subtropical climate of North India. To understand the genetic inheritance of hull-less seed traits, two hulled and three hull-less genotypes of *C. pepo* were used to develop segregating populations. Seeds from all the six generations (P_1_, P_2_, F_1_, F_2_, BC_1_P_1_, and BC_1_P_2_) of four crosses were phenotyped at maturity. The F_1_ plants in all cross combinations developed hulled seeds indicating recessive action of the gene(s) governing hull-less seed trait. Furthermore, chi-square analysis suggested that the hull-less phenotype fit in the Mendelian ratio of 3:1 (hulled: hull-less) in F_2_ and 1:1 in backcross populations (Table 1). These results conclude that the hull-less seed trait is conferred by a single recessive gene in *C. pepo* lines adapted to sub-tropical climate.

**Table 1:** Segregation of hulled and hull-less seed trait in different crosses.

**Generations**	**Hulled seed**	**Hull-less seed**	**Best fit ratio**	**χ^2^ cal**	***p*-value**
Cross combination-I: HP111 × HLP36
F_1_	10	0			
F_2_	392	114	3:1	1.65	0.20
BC_1_P_1_	263	0	–		
BC_1_P_2_	135	111	1:1	2.34	0.13
Cross combination-II: HP111 × HLP53
F_1_	10	0			
F_2_	126	44	3:1	0.07	0.79
BC_1_P_1_	112	0	–		
BC_1_P_2_	64	57	1:1	0.40	0.53
Cross combination-III: HP112 × HLP8
F_1_	10	0			
F_2_	112	32	3:1	0.59	0.44
BC_1_P_1_	105	0	–		
BC_1_P_2_	46	57	1:1	1.17	0.28
Cross combination-IV: HLP8 × HP112
F_1_	10	0			
F_2_	171	51	3:1	0.49	0.48
BC_1_P_1_	71	59	1:1	1.11	0.29
BC_1_P_2_	113	0	–		

### Construction and sequencing of bulks

On the basis of the genetic inheritance pattern of the hull-less seed trait, two extreme bulks, which represent hulled seeds (B_1_) and hull-less seeds (B_2_), were constituted by pooling the DNA of 14 plants each from the F_2:3_ population of HP111 (P_1_, hulled) × HLP36 (P_2_, hull-less) (Figure 1). These two DNA samples along with their parental lines DNA were used to construct libraries and subjected to whole-genome resequencing. A total of 33.00, 37.84, 33.12, and 39.41 million paired-end clean reads were retained for P_1_, P_2_, B_1_, and B_2_, respectively. These trimmed reads were then mapped to the *C. pepo* reference genome. The parents, P_1_ and P_2_, aligned 29 and 34 million reads with a reference genome, yielding an overall alignment rate of 89.05 and 91.98%, respectively. Similarly, a total of 29 and 35 million reads of bulk B_1_ and B_2_ were aligned against the reference genome, which represents the overall alignment rate as 90.87 and 91.19%, respectively. Variant calling in these mapped reads with reference genome resulted in a total of 2,205,961 SNPs/Indels. The filtering of these variants retained 1,150,511 SNPs, which were used for the analysis. Furthermore, these SNPs were filtered in QTLseqr and resulted in 39,827 SNPs, which were used for the QTL(s) analysis in this pipeline.

### QTL-seq predicted candidate genomic region governing hull-less seed trait

**Figure 2:**
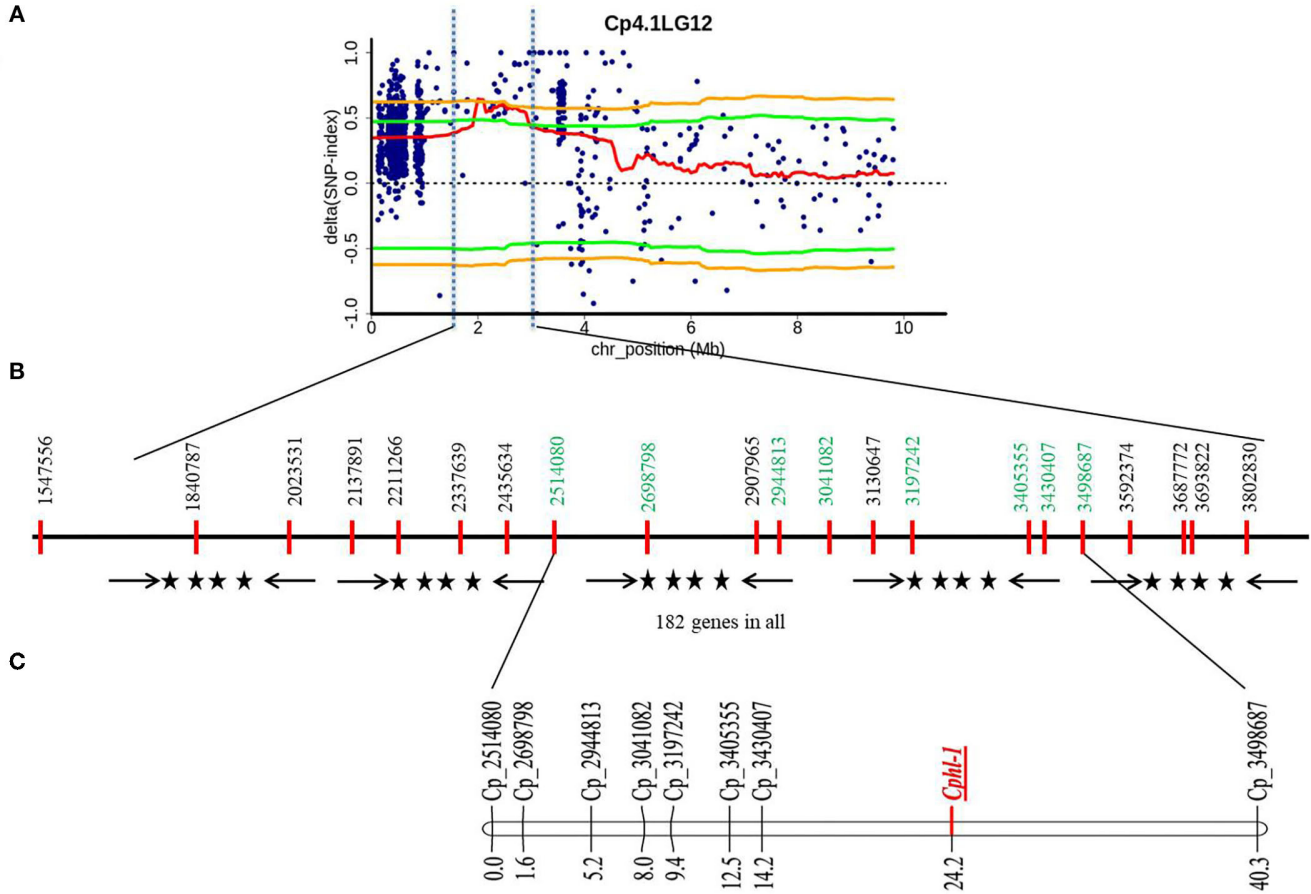
Physical and genetic position of QTL region governing hull-less seed trait in *C. pepo* on chromosome 12. **(A)** ΔSNP index graph from hulled and hull-less bulks, Orange line indicates 99% confidence interval and the green line indicates 95% confidence interval upper/lower side; **(B)** Genomic position of SNPs in QTL region selected for designing KASP assay where SNP shown in green were polymorphic and used for linkage map construction; **(C)** Partial Genetic linkage map of chromosome 12 from the HP111 × HLP36 F_2_ population showing the position of the hull-less locus (*Cphl-1*).

QTL-Seq pipeline (Takagi et al., 2013) was used to identify the candidate genomic region governing hull-less seed trait in *C. pepo*, in which SNP-index was calculated for each identified SNP by comparing to the reference genome assembly in both the bulks. Then, the average SNP-index of each bulk was computed within a sliding window of 2 Mb interval with a 50 kb increment and was plotted against the genomic positions of *C. pepo*. The highly contrasting patterns of SNP-index graphs for hulled (B_1_) and hull-less bulk (B_2_) were observed on chromosome 12 from 1.80 to 2.90 Mb region (Supplementary Figures 1, 2). However, SNP-index graphs on other chromosomes were identical, suggesting these chromosomes might not be relevant to the phenotypic difference between hulled and hull-less bulks. Plotting of SNP-indices between hulled and hull-less bulks using 95 and 99% confidence intervals identified significant genomic positions governing hull-less seed trait. At a 95% statistical level, only one genomic region spanning 1.10 Mb on chromosome 12 from 1.80 to 2.90 Mb had a ΔSNP index value of 0.6 that was significantly different from 0, indicating this region is associated with phenotypic differences between hulled and hull-less bulks (Figure 2A, Supplementary Figure 3).

**Figure 3:**
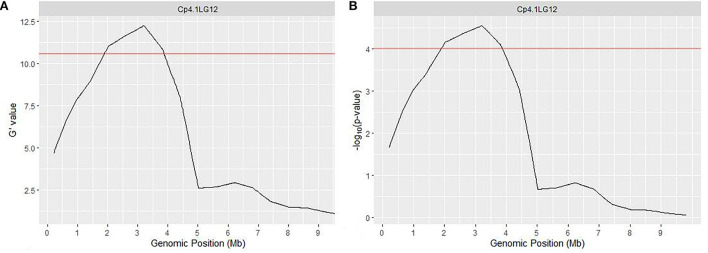
QTL region governing hull-less seed trait in *C*. *pepo* on chromosome 12 using G' **(A)** and *p*-value **(B)**. Red line indicates a significant threshold for FDR (false discovery rate), *q* = 0.01.

QTLseqr pipeline (Mansfeld and Grumet, 2018) also detected a similar genomic region at chromosome 12, spanning 1.89 to 3.86 Mb and covering 1.97 Mb. The peak of ΔSNP index was recorded at 2,615,628 bp at a 99% confidence interval (Supplementary Figure 4), whereas the maximum *G*' value was predicted at 3,209,540 bp w.r.t FDR (*q*) of 0.01 (Figure 3A, Supplementary Figure 5). Furthermore, the *p*-value analysis revealed a significant peak on chromosome 12 with a mean *p*-value of 4.98e^−05^ w.r.t FDR (*q*) of 0.01 (Figure 3B, Supplementary Figure 6). Overall, these results indicated a major QTL related to the hull-less seed trait at chromosome 12 from 1.80 to 3.86 Mb region.

### Molecular mapping of a hull-less locus

**Figure 4:**
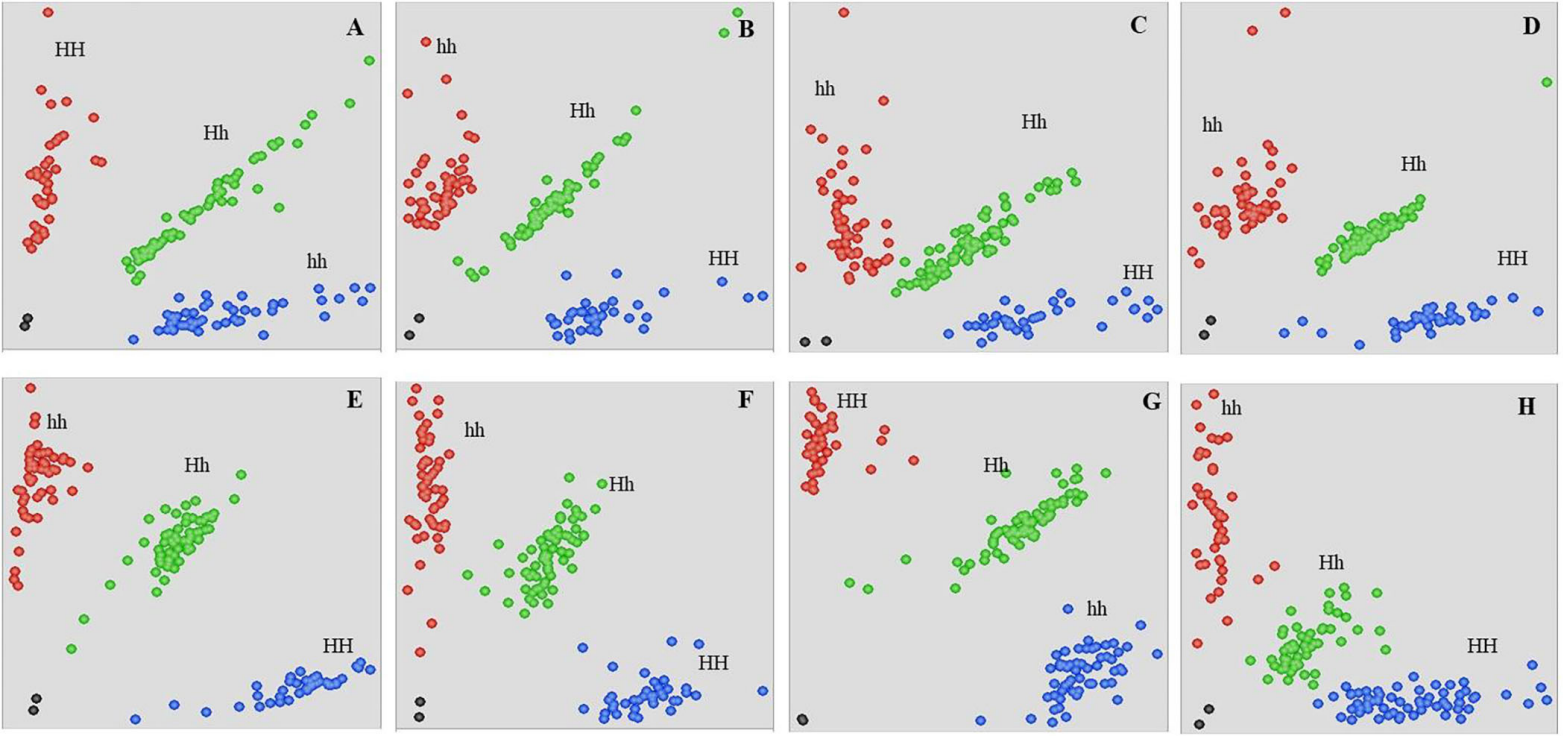
Genotyping of HP111 × HLP36 derived 160 F_2_ individuals using the KASP assay. Scatter plots for KASP assays show clustering of individual plants on the *X*- (FAM) and *Y*- (HEX) axes. Blue and red dots represent the homozygous plants, and green dots represent the heterozygous plants. Black dots represent the NTC (non-template control). HH, Homozygous for hulled parent SNP; hh, Homozygous for hull-less parent SNP; Hh, Heterozygous; **(A)** Cp_2514080; **(B)** Cp_2698798, **(C)** Cp_2944813, **(D)** Cp_3041082, **(E)** Cp_3197242, **(F)** Cp_3405355, **(G)** Cp_3430407, **(H)** Cp_3498687.

To validate QTL-seq results and to further narrow down the candidate region, 21 KASP assays were designed (Figure 2B). However, only eight KASP assays scored reliably on the parents, F_1_ as well as in the hulled and hull-less bulks (Supplementary Figure 7). These polymorphic markers were used to genotype 160 F_2_ plants derived from the cross HP111 × HLP36 to study marker trait association for hull-less seed (Figure 4). The KASP assays were able to accurately predict hull-less plants, ranging from 73.47% to 87.23% of the F_2_ individuals (Table 2). The hull-less locus, *Cphl-1* (named as *Cucurbita pepo* hull-less), was mapped to the partial linkage map of 40.3 cM with closest flanking markers *viz*., Cp_3430407 and Cp_3498687 placed at 10.0 cM and 16.1 cM genetic distances from *Cphl-1* locus, respectively (Figure 4C). The physical locations of these two markers on chromosome 12 of the *C. pepo* genome were 3430407 bp (Cp_3430407) and 3498687 bp (Cp_3498687), spanning 68.28 kb.

**Table 2:** Marker-phenotype association in amplified F_2_ individuals of HP111 × HLP36 cross.

**Sr. No**.	**Marker**	**Phenotype**	**Accuracy prediction**			**Overall accuracy**
			**Genotypic**	**Phenotypic**	**Percent**	**(%)**
1	Cp_2514080	Hull-less	36	49	73.47	84.31
		Hulled	93	104	89.42	
2	Cp_2698798	Hull-less	37	47	78.72	85.91
		Hulled	91	102	89.22	
3	Cp_2944813	Hull-less	37	48	77.08	85.71
		Hulled	95	106	89.62	
4	Cp_3041082	Hull-less	41	47	87.23	90.26
		Hulled	98	107	91.59	
5	Cp_3197242	Hull-less	35	41	85.37	89.29
		Hulled	90	99	90.91	
6	Cp_3405355	Hull-less	39	45	86.67	87.92
		Hulled	92	104	88.46	
7	Cp_3430407	Hull-less	40	46	86.96	90.45
		Hulled	101	109	92.66	
8	Cp_3498687	Hull-less	36	46	78.26	90.67
		Hulled	100	104	96.15	

**Figure 5:**
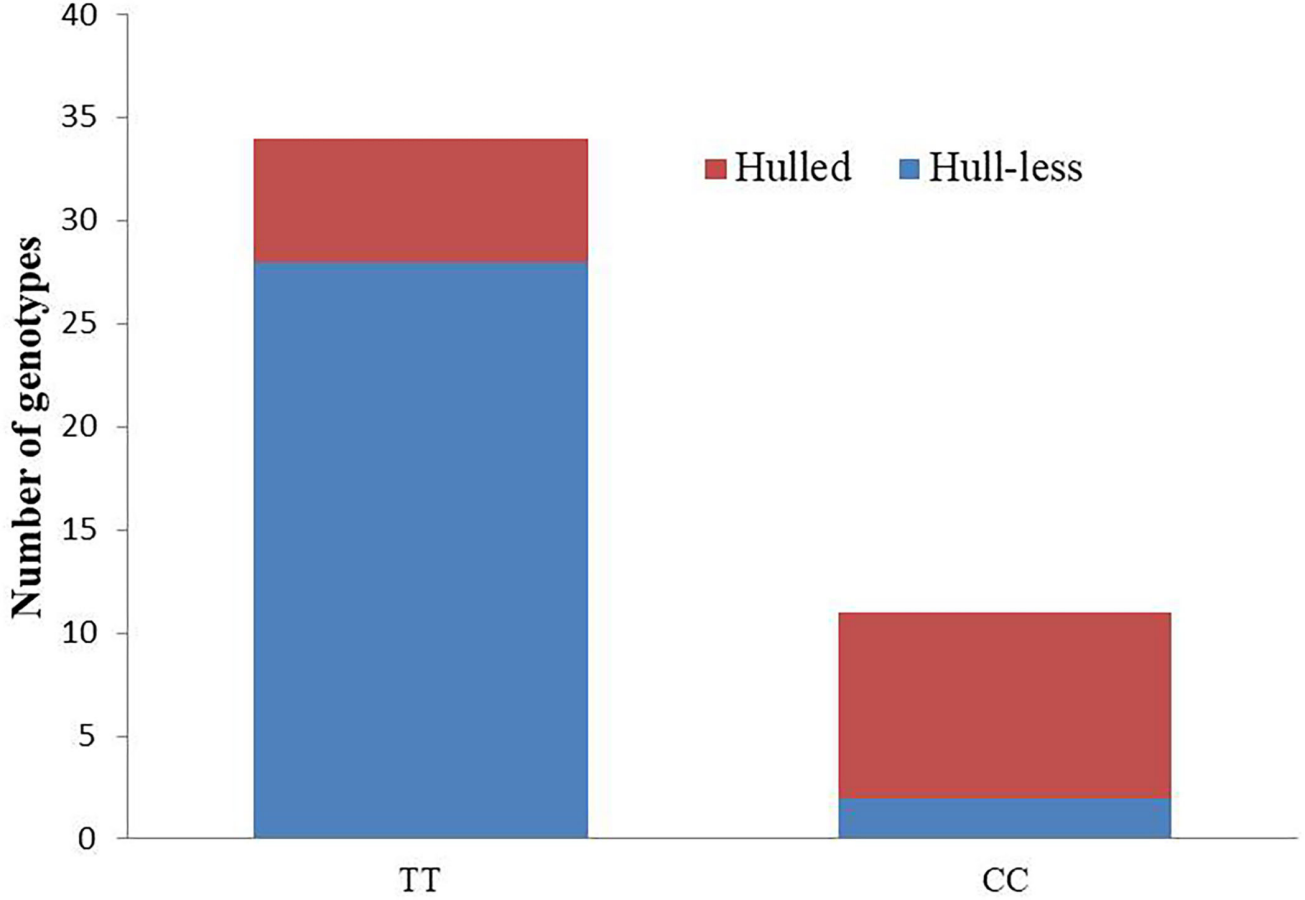
Genotypic and phenotypic data for KASP assay, Cp_3430407 in the diversity panel. (Total number of genotypes = 45; hull-less: 30; hulled: 15); *X*-axis indicates the genotype of Cp_3430407 KASP assay (TT or CC), and *Y*-axis indicates a number of genotyped individuals. Red and Blue sections denote the number of individuals with hulled and hull-less seed types, respectively.

Furthermore, based on the linkage map, two KASP assays (Cp_3430407 and Cp_3498687) were selected to determine their potential in differentiating hull-less and hulled seeds in genetically diverse populations. Marker Cp_3430407 precisely predicted the phenotype in the evaluated hull-less genotypes except two (28 out of 30), giving an accuracy percentage of 93.33. However, the accuracy was reduced to 60% in hulled genotypes as only nine out of 15 hulled genotypes were accurately predicted (Figure 5). Thus, the results indicate that the marker Cp_3430407 can be used in marker-assisted selection for hull-less seed trait till the gene-based marker can be identified.

### Candidate gene identification, functional annotation, transcriptional differences, and gene ontology analysis

**Figure 6:**
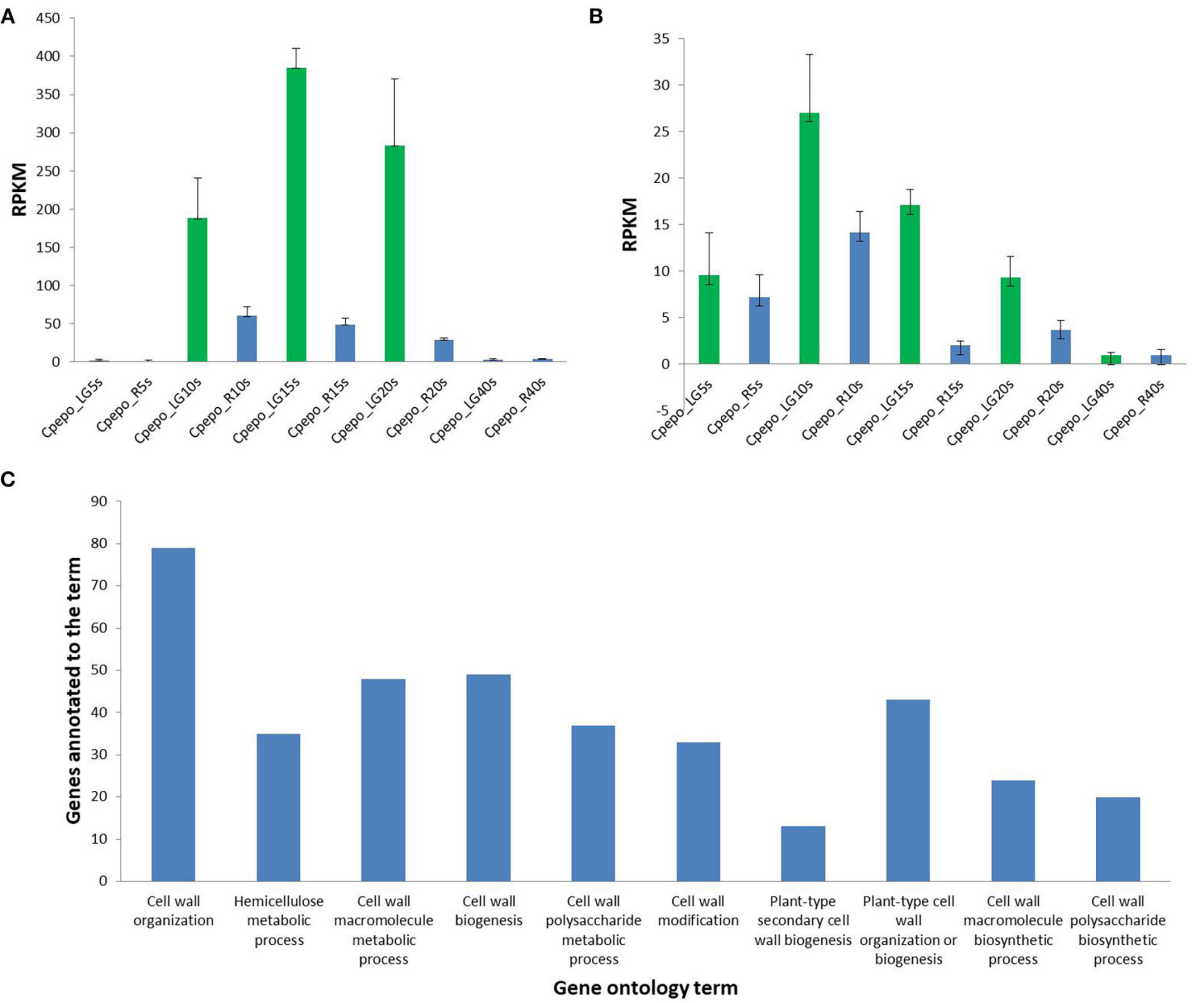
Differential expression of genes, NAC (*Cp4.1 LG12g04350*) **(A)** and MYB, (*Cp4.1LG12g03120*) **(B)** in seeds of hull-less and hulled *C. pepo*; where, RPKM, Reads per Kilobase of transcript per million mapped read; LG, hull-less seeded variety “Lady Godiva”; R, Hulled variety “Sweet Reeba”; 5, 10, 15, 20, and 40 s corresponds to 5, 10, 15, 20, and 40 DAP **(C)**: Gene ontology analysis (Biological processes) from transcriptional differences between hull-less and hulled seeds at 15 and 20 DAP.

The candidate genomic region associated with the hull-less seed trait in *C. pepo* consists of 182 genes (Supplementary Table 2), out of which 19 are directly or indirectly involved in the cell wall, cellulose, and lignin biosynthesis (Table 3). The expression of these 19 genes in seeds of hulled and hull-less *C. pepo* genotypes assessed from the publically available RNA-seq dataset revealed two genes *viz*., *Cp4.1LG12g04350* (NAC) and *Cp4.1LG12g03120* (MYB), which are highly expressed in hull-less genotype compared to the hulled genotype at different time intervals (Figures 6A,B; Supplementary Table 3). These genes share homology with the *Arabidopsis* secondary cell wall related NAC and MYB transcriptional factors, respectively, which act as master regulators of lignin biosynthesis (Zhong and Ye, 2009). Furthermore, the differential expression analysis at 15 and 20 DAP between seeds of hulled and hull-less genotypes found a total of 3,124 unique differentially expressed genes. The gene ontology analysis (biological process) of these genes linked 482 to the cell wall, cellulose, or lignin biosynthesis that corresponds to 15.42% of the total (Figure 6C). These results imply that the transcriptional profile of genes involved in cell wall development and lignification was altered between hulled and hull-less seeds.

**Table 3:** List of genes associated with the cell wall, lignin, or cellulose biosynthesis from the candidate region governing hull-less seed trait.

**Sr. No**.	**Gene**	**Protein**	**Start**	**End**	**Blast hit on TAIR 10**	**Function**
1	Cp4.1LG12g02800	UDP-glucuronate decarboxylase protein 1	1,877,593	1,881,852	AT3G62830	Substrate for many cell wall carbohydrates (hemicellulose and pectin)
2	Cp4.1LG12g02990	BnaA02g30570D protein	1,895,289	1,897,094	AT5G49100	Cell wall biogenesis, plant-type cell wall organization or biogenesis
3	Cp4.1LG12g03090	Beta xylosidase	2,045,702	2,051,382	AT5G49360	Gene is expressed specifically in tissues undergoing secondary wall thickening
4	Cp4.1LG12g03100	Dihydrolipoyl dehydrogenase	2,073,784	2,080,210	AT3G16950	The gene is highly expressed in developing seeds
5	Cp4.1LG12g03120	MYB transcriptional factor	2,030,099	2,031,025	AT4G38620	Cell wall biogenesis
6	Cp4.1LG12g03440	Polygalacturonase	2,295,190	2,299,042	AT1G48100	Plant-type cell wall modification
7	Cp4.1LG12g03640	Glycerol-3-phosphate acyltransferase	2,673,157	2,676,758	AT4G00400	Cutin biosynthetic process
8	Cp4.1LG12g03660	Signal peptide peptidase-like protein	2,648,584	2,657,840	AT1G63690	Cell wall organization or biogenesis
9	Cp4.1LG12g03680	Myc anthocyanin regulatory protein	2,613,869	2,621,177	AT1G32640	Regulates the expression of NST1
10	Cp4.1LG12g03720	Cytochrome P450 family protein	2,678,868	2,680,523	AT2G45970	Seed coat development
11	Cp4.1LG12g03790	Leucine-rich repeat/extensin	2,744,480	2,747,086	AT4G13340	Involved in cell wall biogenesis and organization
12	Cp4.1LG12g04010	Xyloglucan galactosyltransferase KATAMARI1	2,900,208	2,903,680	AT2G20370	Synthesis of cell wall materials
13	Cp4.1LG12g04020	G-box binding, MFMR	2,888,085	2,888,924	AT3G08600	Cell wall organization or biogenesis
14	Cp4.1LG12g04040	Long-chain-fatty-acid CoA ligase, putative	2,919,014	2,934,658	AT2G47240	Cutin biosynthetic process, wax biosynthetic process
15	Cp4.1LG12g04070	Receptor-like kinase	2,941,245	2,944,833	AT3G23750	Cell wall biogenesis
16	Cp4.1LG12g04350	NAC domain-containing protein, putative	3,411,999	3,413,380	AT2G46770	Plant-type secondary cell wall biogenesis
17	Cp4.1LG12g04370	Protein IQ-DOMAIN 14	3,406,540	3,408,651	AT4G00820	Plant-type cell wall organization or biogenesis
18	Cp4.1LG12g04380	Basic helix loop helix (BHLH) family transcription factor	3,393,961	3,396,917	AT1G32640	Regulation of secondary cell wall biogenesis
19	Cp4.1LG12g04400	Auxin-responsive family protein	3,679,140	3,679,442	AT5G53590	Cell wall organization or biogenesis

## Discussion

Pumpkin (*C. pepo*) seeds have a high nutritive value that imparts numerous health benefits (Dotto and Chacha, 2020). However, these are covered by a thick and leathery seed coat due to the lignification of different cell wall layers, and therefore, require its removal prior to use for human consumption. On the contrary, hull-less pumpkin mutant, such as Styrian, lacks the hard seed coat and, as a result, it does not require de-hulling. Hull-less seed trait was transferred from a USA cultivar, “Lady Godiva” into local *C*. *pepo* lines adapted to the subtropical climate of India. Genetic inheritance in different hulled and hull-less populations suggested that lack of seed coat lignification is governed by a single recessive gene in *C. pepo* lines. In the past, different studies in European germplasm demonstrated that a single recessive gene (*hh*) is responsible for the hull-less seed trait in pumpkin. Although hull-less seeds exhibit variation for a degree of lignification, which might be governed by unspecified minor genes (Lelley et al., 2009).

The selection for hulled and hull-less seeds at the phenotypic level is performed after the maturation of fruits on a visible, sensory, and microscopic basis, but these methods are very time consuming and labor intensive. These limitations can be overcome by developing molecular markers that breeders can use in marker-assisted breeding programmes in a cost and time-effective manner (Collard and Mackill, 2008). Bulk segregant analysis (BSA) is a rapid method to identify markers linked to the trait of interest (Michelmore et al., 1991) and when combined with NGS technologies can be used as a fast-track approach to locate candidate genomic regions more rapidly. This approach is known as QTL-seq and involves the selection of extreme phenotypic values, pooling in equivalent concentration, followed by sequencing of pools for downstream sequence analysis in different crops (Takagi et al., 2013; Bhat et al., 2016; Li and Xu, 2022). The utilization of this approach accelerated the identification of genomic regions and candidate genes governing traits of economic importance and the development of molecular markers that could be used in MAS (Grover and Sharma, 2016; Tan et al., 2018). The availability of the *C. pepo* genome opens up new avenues to discover the genomic region controlling hull-less seed trait.

In this study, we utilized NGS-based BSA of hulled and hull-less genotypes followed by analysis with QTL-seq and QTLseqr bioinformatics pipelines to determine the genomic region responsible for hull-less seed in *C. pepo*. The power of the QTL-seq method typically depends upon population size and proportion used in the bulk generation. In general, bulk sizes of 15–20% from a F_2_ population of 200–300 individuals are considered sufficient to detect both major and minor QTLs (Magwene et al., 2011; Takagi et al., 2013; Tiwari et al., 2016). However, a single major QTL can be detected using an F_2_ population of 100 individuals (Takagi et al., 2013; Illa-Berenguer et al., 2015). BSA coupled with the NGS approach has been extensively used in the mapping of major effect traits controlled by one or two loci. Thus, this is the effective approach for mapping hull-less loci, as it is governed by a single recessive gene. Moreover, the various mapping studies reported the bulk sizes in the range of 3–11.9% (Takagi et al., 2013; Singh et al., 2016; Wei et al., 2016; Ramos et al., 2020). The filial populations produced through selfing can significantly increase the power and precision, especially from F_2_ to F_3_, and have been used in the present study (Huang et al., 2022).

The hull-less locus was mapped on chromosome 12 with a genomic region extending from 1.80 to 3.86. Earlier, the hull-less seed locus was mapped to the linkage group LG09 (Gong et al., 2008); however, its physical position was not known. The BLAST results of the linked SSR markers (CMTm239, CMTp182) from this study revealed significant hits on chromosome 12 of the *C. pepo* genome, as of the identified region in our study. While we were preparing this manuscript, Meru et al. (2022) identified a large genomic region of 4.43 Mb (1.25–5.68 Mb) on chromosome 12 for the hull-less seed trait; however, in our study, it was narrowed down to ~2 Mb (1.80–3.86 Mb), which might be due to higher number of individuals used for bulk construction.

Over the years, SNPs-based markers have become the first choice for researchers due to their high genomic abundance, co-dominant inheritance, high-throughput analysis, and relatively low genotyping error rates (Mammadov et al., 2012). Among the different platforms, the KASP assay is a promising technology for high throughput SNP genotyping (Ayalew et al., 2019). The SNP-based KASP assays for MAS have been developed for many traits in cucurbits (Paudel et al., 2019; Cao et al., 2021; Kahveci et al., 2021). The polymorphic KASP assays have been used in the present investigation to develop a partial genetic linkage map of 40.3 cM and the *Cphl-1* locus was delimited between two markers Cp_3430407 and Cp_3498687. A KASP assay *viz*., Cp_3430407 can be utilized for MAS with 93.33% accuracy in hull-less germplasm of *C. pepo*. The physical positions of this KASP assay are also close to recently identified SNP markers (Ch12_3412046 and Ch12_3417142) reported by Meru et al. (2022).

The molecular mechanism of cell wall lignification has been well-studied in *Arabidopsis*, and over the years, this knowledge has been translated to understand the lignification mechanism in crops (Wang and Dixon, 2012; Barros et al., 2015). In this study, we identified a candidate genomic region associated with hull-less seed trait in *C. pepo* that tends to have limited lignification on seed coat in comparison to hulled seeds. Furthermore, two *C. pepo* genes from this region share homology with NAC and MYB transcription factors which act as master regulators of lignification (Wang et al., 2011; Yoon et al., 2015). These genes were highly expressed in hull-less seeds between 10 and 20 days after anthesis in comparison to hulled genotypes (Wyatt et al., 2015). Similarly, a high proportion of cell wall related genes (15.42%) was differentially expressed between hulled and hull-less seeds at 15 and 20 DAP. Based on earlier studies, it has been demonstrated that the extent of lignification starts increasing in hulled seeds 10 days after pollination in comparison to hull-less seeds (Bezold et al., 2005; Xue et al., 2022). In the future, it will be fascinating to study the functional role of candidate genes differentially expressed between hulled and hull-less seeds of pumpkin to understand the molecular mechanism of seed coat lignification.

## Conclusion

Hull-less seed trait in pumpkin has shown monogenic recessive inheritance in *C. pepo*. QTL-seq analysis has also shown a single major QTL on chromosome 12 spanning 2.06 Mb associated with hull-less seed, which was further delimited to ~68 kb using KASP assays. Further analysis of candidate genes spanning the QTL region has identified lignin-related genes, NAC, and MYB as potential candidate genes governing hull-less seed trait in *C. pepo*. Finally, the linked KASP assay generated in the current investigation can further be used in breeding programmes for marker-assisted selection of hull-less trait. The study has also laid the foundation for research on molecular mechanisms related to hull-less seed trait in pumpkin.

## Data availability statement

The datasets presented in this study can be found in online repositories. The names of the repository/repositories and accession number(s) can be found below: https://www.ncbi.nlm.nih.gov/sra/PRJNA849145.

## Author contributions

AD conceived the research. AD, DB, JK, AM, and MS conceptualized the experiments. BK and KG conducted experiments and analyzed the data. All authors have prepared and approved the final manuscript.

## Conflict of interest

The authors declare that the research was conducted in the absence of any commercial or financial relationships that could be construed as a potential conflict of interest.

## Publisher's note

All claims expressed in this article are solely those of the authors and do not necessarily represent those of their affiliated organizations, or those of the publisher, the editors and the reviewers. Any product that may be evaluated in this article, or claim that may be made by its manufacturer, is not guaranteed or endorsed by the publisher.

## Supplementary material

Supplementary Figure 1 SNP-index distribution for hulled bulk sample (B_1_). Green dots: SNP-index of hulled bulk B_1_'s; Red line: Sliding window average of SNP-index.

Supplementary Figure 2 SNP-index distribution for hull-less bulk sample (B_2_). Orange dot: SNP-index of hull-less bulk B_2_'s; Red line: Sliding window average of SNP- index.

Supplementary Figure 3 ΔSNP-index distribution in hulled and hull-less bulks. Blue dots: ΔSNP-index; Red line: Sliding window average of ΔSNP-index; Green line: Sliding window average of 95%-confidence interval upper/lower side; Orange line: Sliding window average of 99%-confidence interval upper/lower side.

Supplementary Figure 4 Tricube smoothed ΔSNP-index distribution across 20 chromosome of *C. pepo*. Red line: 95% confidence interval; Blue line: 95% confidence interval**;** *X*-axis: Physical position (Mb) of C. pepo genome cv. Zucchini across the respective chromosome number; *Y*-axis: Designates the ΔSNP-index.

Supplementary Figure 5 G' distribution across 20 chromosomes of *C. pepo*. Red line: Significant threshold for FDR (false discovery rate), *q*=0.01; X-axis: Physical position (Mb) of C. pepo genome cv. Zucchini across the respective chromosome number; Y-axis: Designates the G' value.

Supplementary Figure 6 *p*-value distribution across 20 chromosomes of *C. pepo*. Red line: Significant threshold for FDR (false discovery rate), *q* = 0.01; *X*-axis: Physical position (Mb) of C. pepo genome cv. Zucchini across the respective chromosome number; *Y*-axis: Designates the -log10 (*p*-value).

Supplementary Figure 7 Genotyping of parents, F_1_, and bulk individual F_2:3_ lines using the Kompetitive Allele Specific PCR (KASP) assay. Scatter plots for selected KASP assays show clustering on the *X*- (FAM) and *Y*-axes (HEX). The blue and red dots represent the homozygous bulk individuals, and the green dots represent heterozygous plants. The black dots represent the NTC (non-template control). The pink dots represent the non-amplified plants. HH, Homozygous for hulled parent SNP; hh, Homozygous for hull-less parent SNP; Hh, Heterozygous; **(A)** Cp_2514080, **(B)** Cp_2698798, **(C)** Cp_2944813, **(D)** Cp_3041082, **(E)** Cp_3197242, **(F)** Cp_3405355, **(G)** Cp_3430407, **(H)** Cp_3498687.

Supplementary Table 1 List of KASP markers used in the study.

Supplementary Table 2 List of genes present in the candidate region.

Supplementary Table 3 Variance for RNA-seq expression between seeds of hull-less and hulled genotypes at different time intervals.
