# Supplementary figures and images for: Identification of single major QTL and candidate gene(s) governing hull-less seed trait in pumpkin
Source: Front Plant Sci. 2022 Aug 11;13:948106. doi: 10.3389/fpls.2022.948106 (PMC9406289; doi:10.3389/fpls.2022.948106)

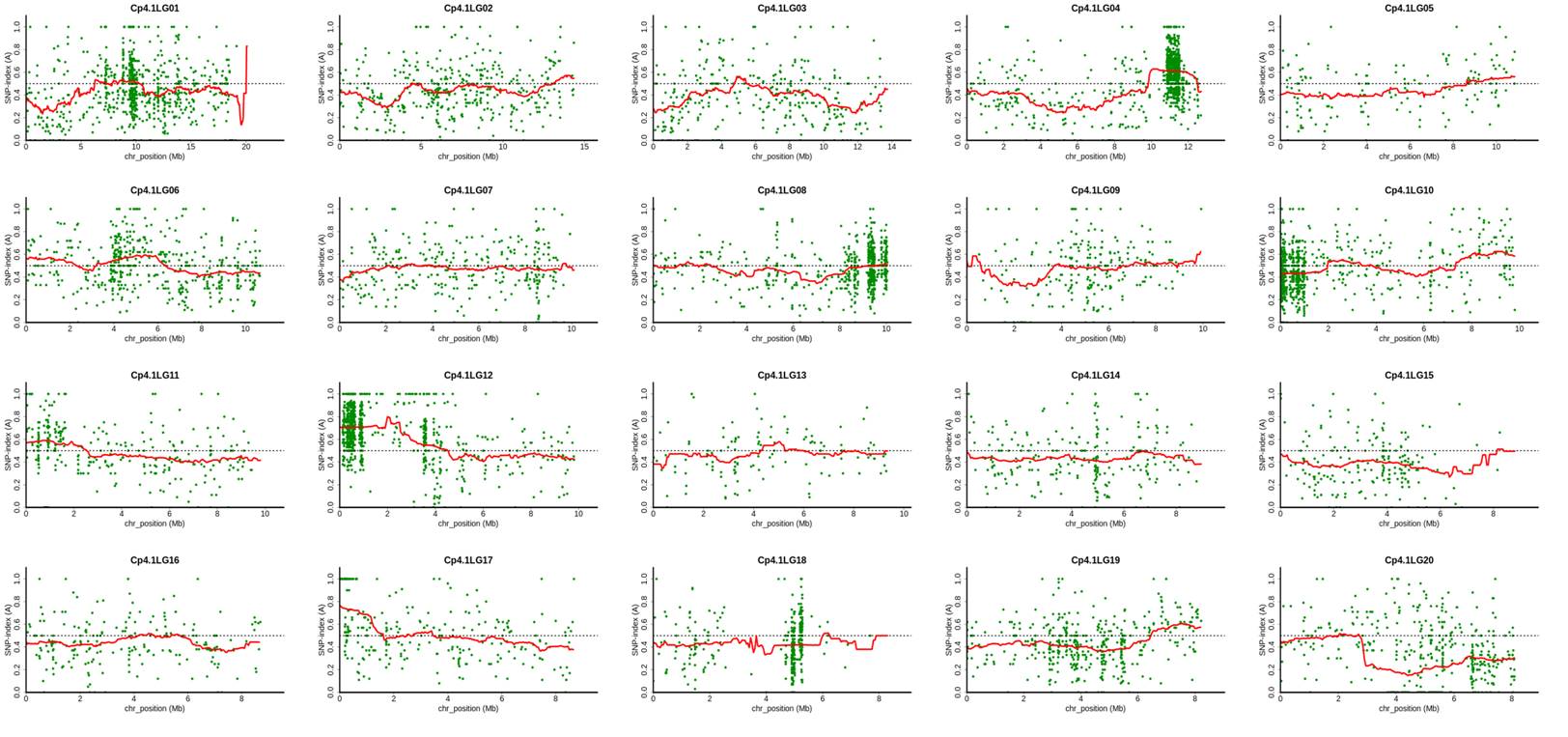

Supplement: Supplementary Figure 1 — SNP-index distribution for hulled bulk sample (B1). Green dots: SNP-index of hulled bulk B1's; Red line: Sliding window average of SNP-index. [file Image_1.JPEG]

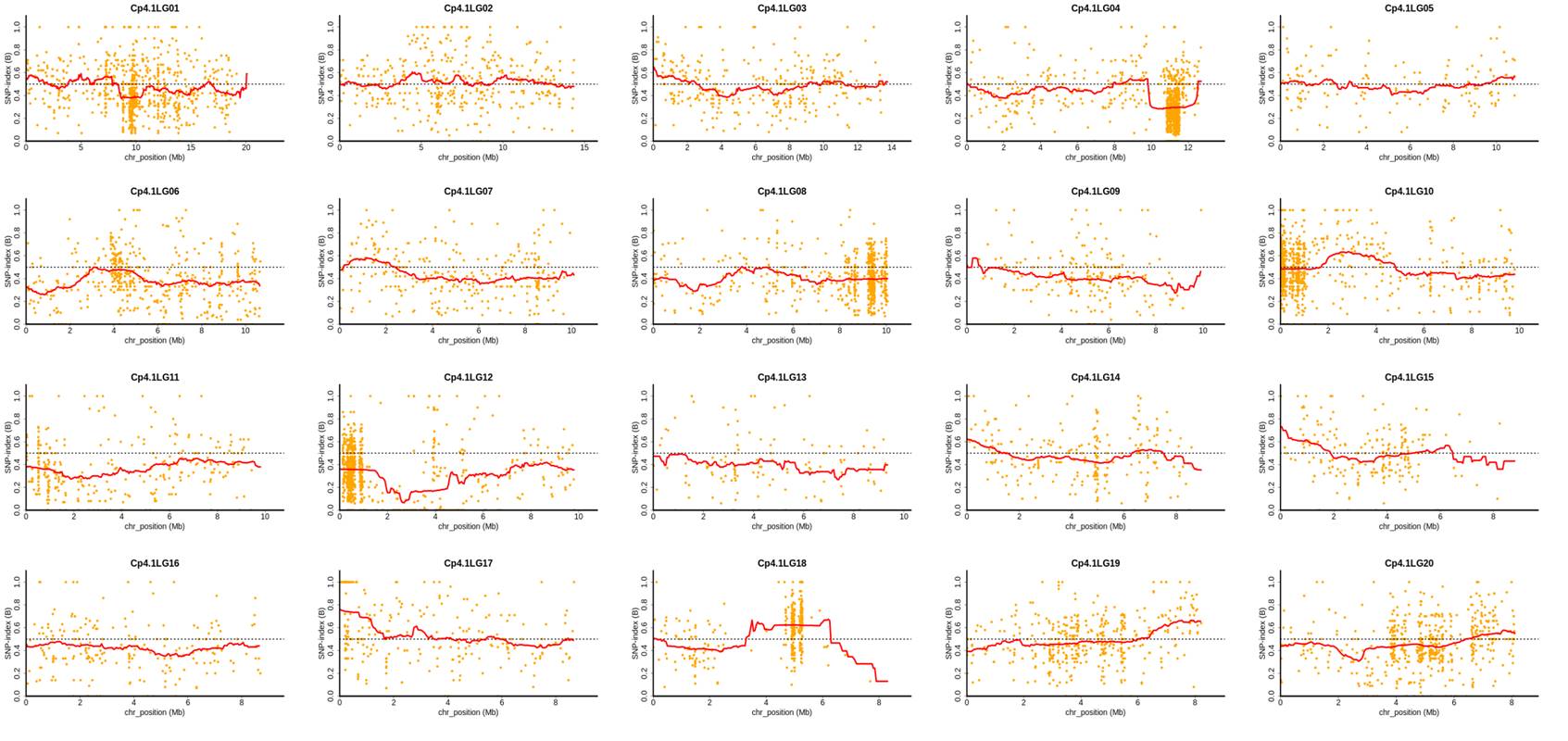

Supplement: Supplementary Figure 2 — SNP-index distribution for hull-less bulk sample (B2). Orange dot: SNP-index of hull-less bulk B2's; Red line: Sliding window average of SNP- index. [file Image_2.JPEG]

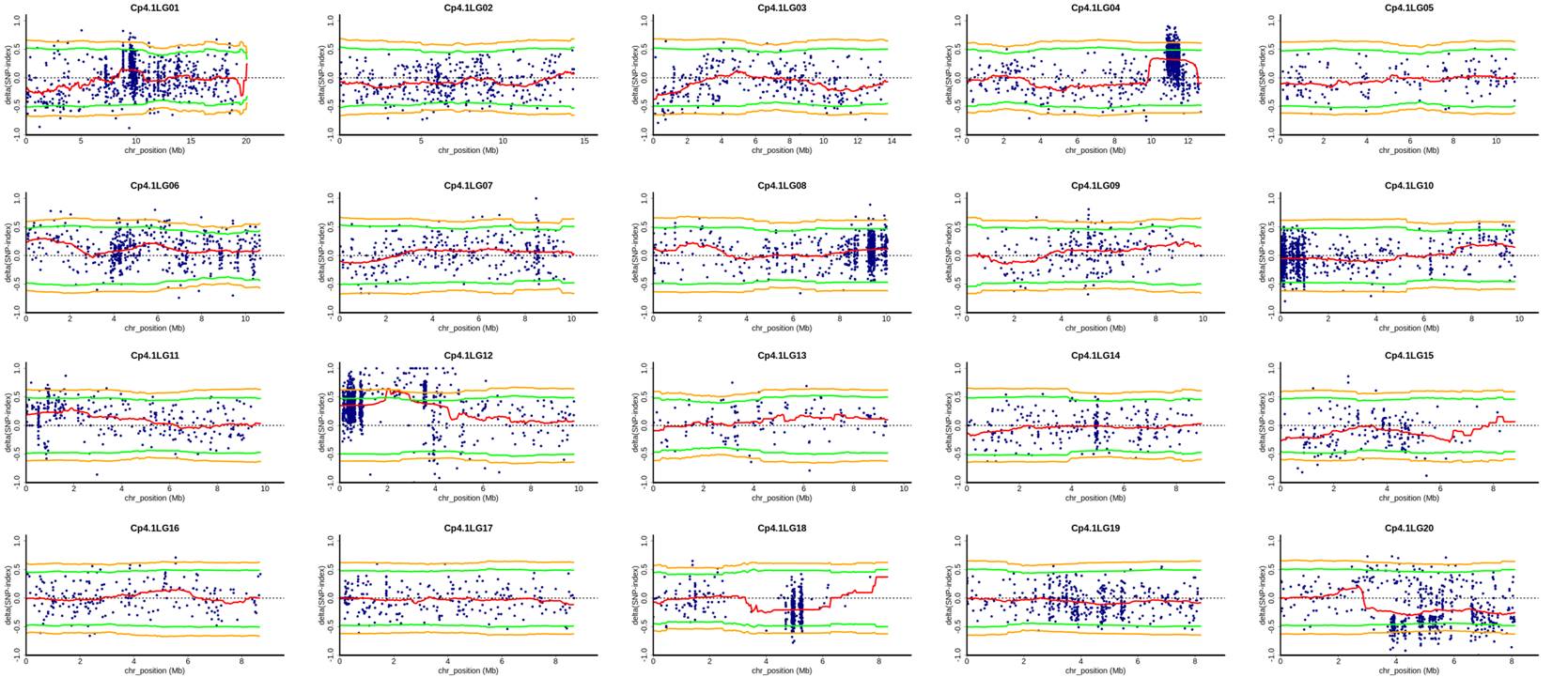

Supplement: Supplementary Figure 3 — ΔSNP-index distribution in hulled and hull-less bulks. Blue dots: ΔSNP-index; Red line: Sliding window average of ΔSNP-index; Green line: Sliding window average of 95%-confidence interval upper/lower side; Orange line: Sliding window average of 99%-confidence interval upper/lower side. [file Image_3.JPEG]

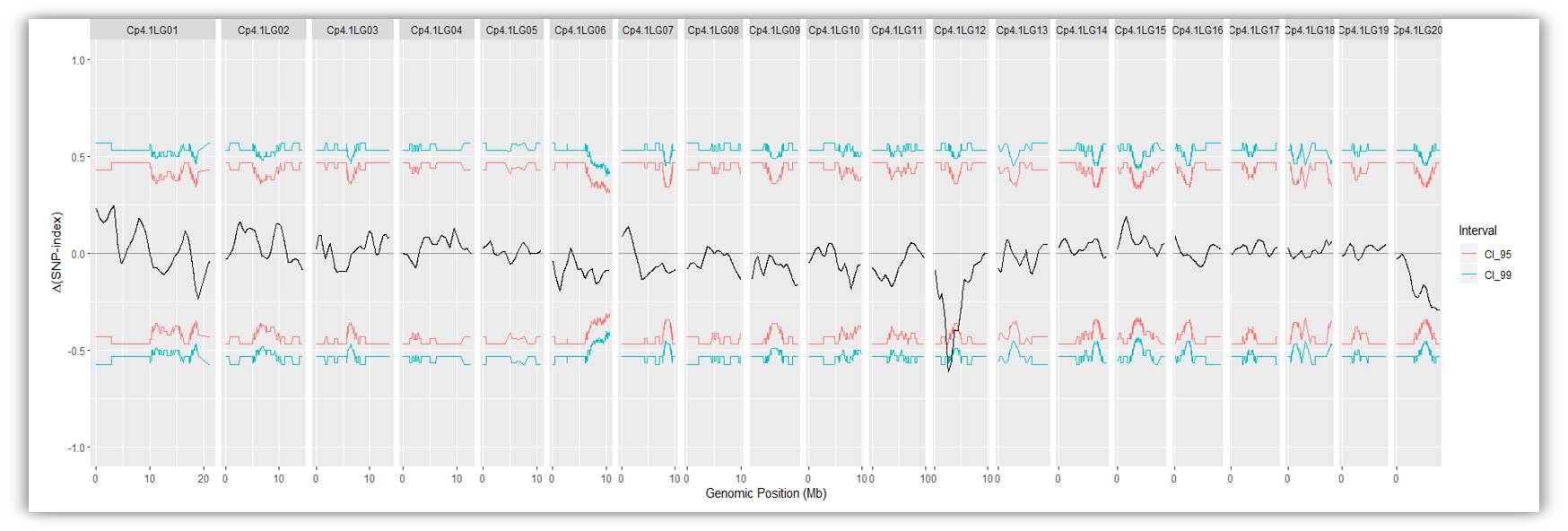

Supplement: Supplementary Figure 4 — Tricube smoothed ΔSNP-index distribution across 20 chromosome of C. pepo. Red line: 95% confidence interval; Blue line: 95% confidence interval; X-axis: Physical position (Mb) of C. pepo genome cv. Zucchini across the respective chromosome number; Y-axis: Designates the ΔSNP-index. [file Image_4.JPEG]

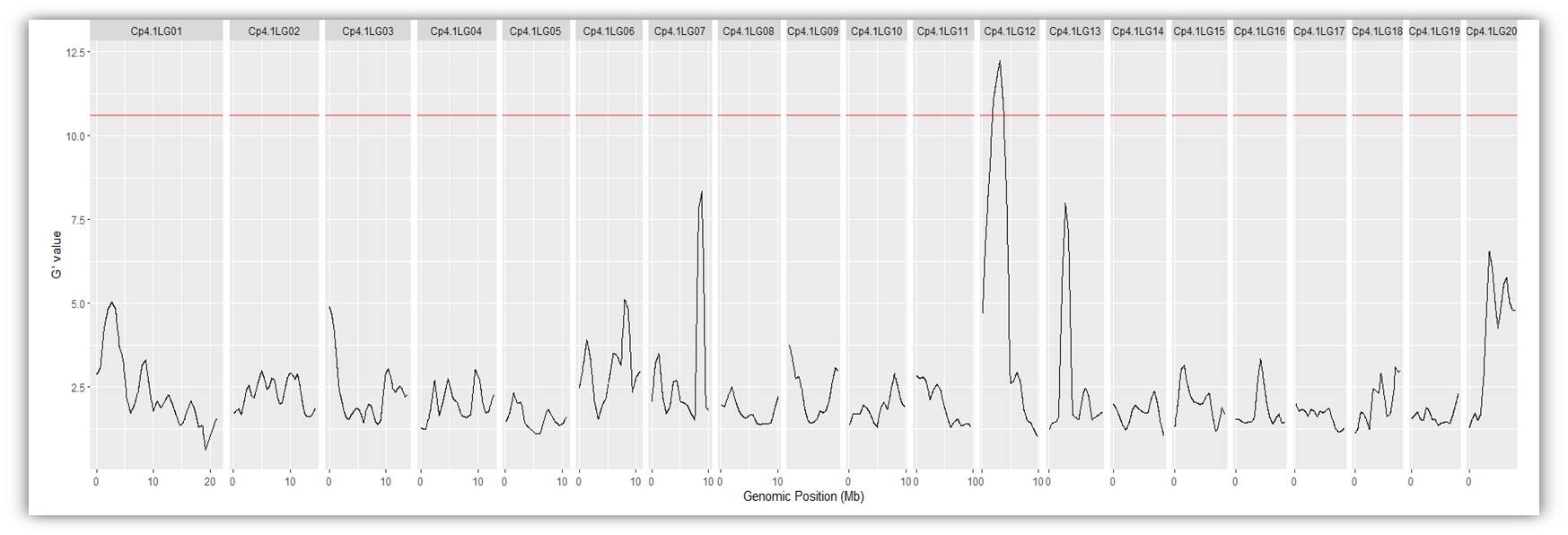

Supplement: Supplementary Figure 5 — G' distribution across 20 chromosomes of C. pepo. Red line: Significant threshold for FDR (false discovery rate), q=0.01; X-axis: Physical position (Mb) of C. pepo genome cv. Zucchini across the respective chromosome number; Y-axis: Designates the G' value. [file Image_5.JPEG]

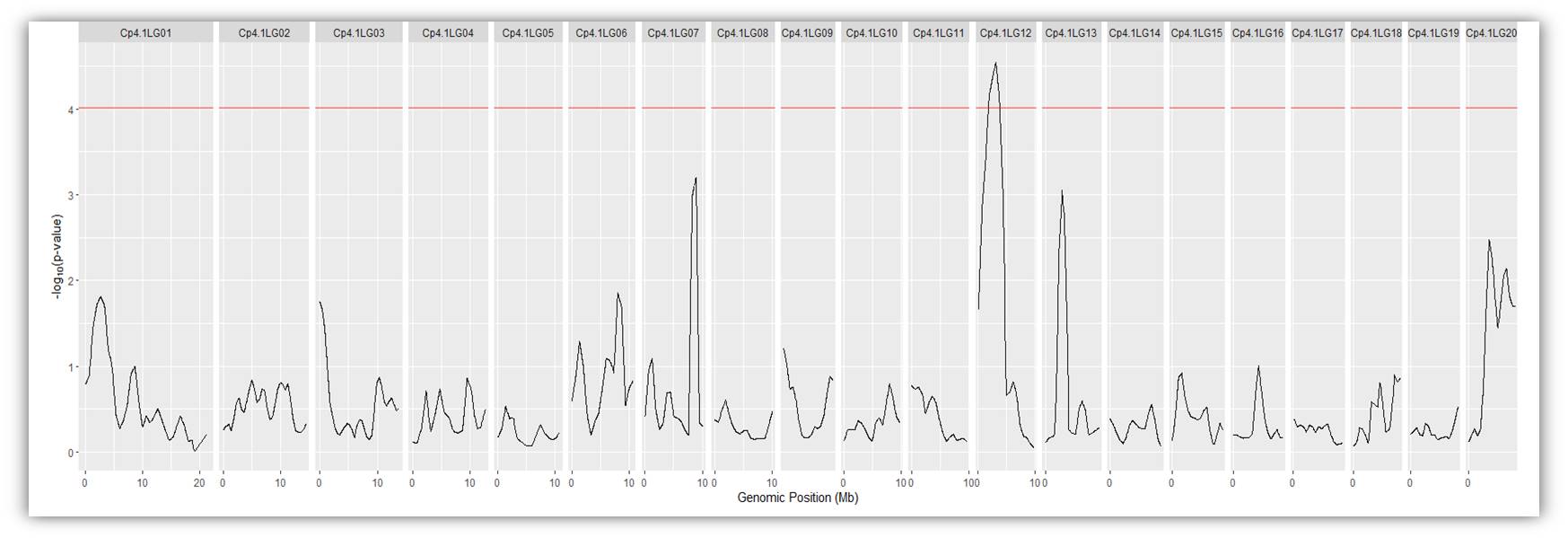

Supplement: Supplementary Figure 6 — p-value distribution across 20 chromosomes of C. pepo. Red line: Significant threshold for FDR (false discovery rate), q = 0.01; X-axis: Physical position (Mb) of C. pepo genome cv. Zucchini across the respective chromosome number; Y-axis: Designates the -log10 (p-value). [file Image_6.JPEG]

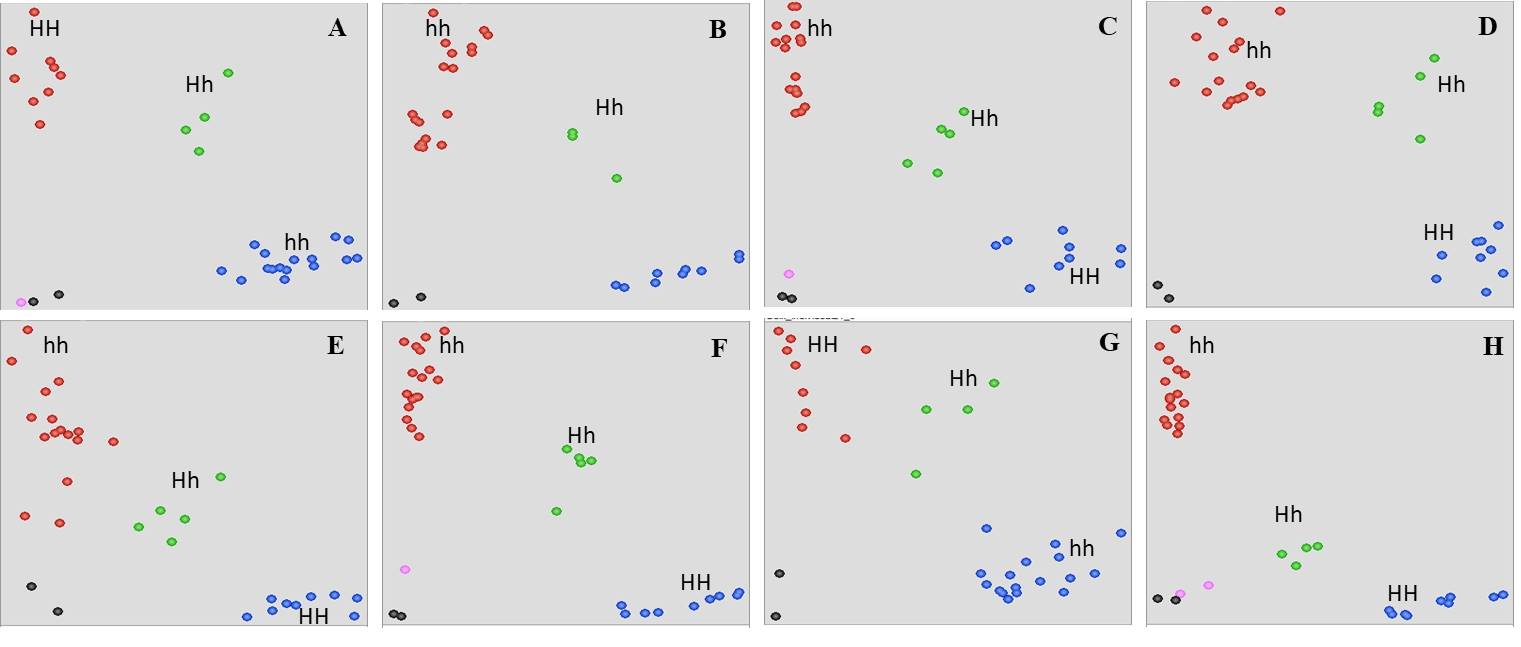

Supplement: Supplementary Figure 7 — Genotyping of parents, F1, and bulk individual F2:3 lines using the Kompetitive Allele Specific PCR (KASP) assay. Scatter plots for selected KASP assays show clustering on the X- (FAM) and Y-axes (HEX). The blue and red dots represent the homozygous bulk individuals, and the green dots represent heterozygous plants. The black dots represent the NTC (non-template control). The pink dots represent the non-amplified plants. HH, Homozygous for hulled parent SNP; hh, Homozygous for hull-less parent SNP; Hh, Heterozygous; (A) Cp_2514080, (B) Cp_2698798, (C) Cp_2944813, (D) Cp_3041082, (E) Cp_3197242, (F) Cp_3405355, (G) Cp_3430407, (H) Cp_3498687. [file Image_7.jpg]
